# Assessment of knowledge and practices of healthcare workers towards infection prevention and associated factors in healthcare facilities of West Arsi District, Southeast Ethiopia: a facility-based cross-sectional study

**DOI:** 10.1186/s13690-018-0314-0

**Published:** 2018-11-12

**Authors:** Biniyam Sahiledengle Geberemariyam, Geroma Morka Donka, Berhanu Wordofa

**Affiliations:** 1 Department of Public Health, School of Health Science, Madda Walabu University Goba Referral Hospital, Bale-Goba, Ethiopia; 2 Department of Nursing, School of Health Science, Madda Walabu University Goba Referral Hospital, Bale-Goba, Ethiopia; 3 Department of Nursing and Midwifery, School of Health Science, Addis Ababa University, Addis Ababa, Ethiopia

**Keywords:** Infection prevention, Knowledge, Practice, Healthcare-associated infection, Ethiopia

## Abstract

**Background:**

The prevention of healthcare associated infections is central to the provision of safe, high quality healthcare. Infections acquired in healthcare facilities are a major public health concern, contributing to increased morbidity, mortality, and cost in both developed and developing countries. Although most of these infections can be prevented with relatively inexpensive infection prevention and control measures in many developing countries, in sub-Saharan African healthcare facilities have no effective infection prevention programs. Additionally, there is limited information on healthcare worker infection prevention knowledge and practice in countries such as Ethiopia. The aim of this study was to assess the knowledge and practices of healthcare workers with respect to infection prevention and associated factors in healthcare facilities in southeast Ethiopia.

**Methods:**

A facility-based cross-sectional study design was used to study healthcare workers in the southeast, Ethiopia. Multi-stage sampling was employed to select 680 healthcare workers from 30 randomly selected healthcare facilities. Data was collected using a self-administered structured questionnaire. Descriptive statistics were computed. Multivariable logistic regression was performed to identify factors associated with healthcare workers infection prevention knowledge and practice.

**Results:**

A total of 648 healthcare workers participated in this study, for a response rate of 95.3%. Of these, 53.7% (95% CI: 49.8, 57.4%) and 36.3% (95% CI: 32.4, 40.1%) of the respondents were assessed as knowledgeable and reported safe infection prevention practices respectively. The likelihood of self-reporting safe infection prevention practice significantly increased if healthcare workers had received training (AOR = 5.31; 95% CI: 2.42,11.63) and had infection prevention guidelines available (AOR = 3.34; 95% CI: 1.65, 6.76). Healthcare workers were more likely to have infection prevention knowledge if they worked longer ten years or more (AOR = 3.41; 95% CI: 1.22, 9.55); worked in facilities with infection prevention committees (AOR = 1.78; 95% CI: 1.01, 3.13), had infection prevention guidelines available (AOR = 2.44; 95% CI: 1.45, 4.12); had training (AOR = 5.02; 95% CI: 1.45, 8.59).

**Conclusions:**

Inadequate infection prevention knowledge and unsafe practices were frequent among study participants, reflecting a potentially common problem at public healthcare facilities in southeast Ethiopia. Healthcare workers have better knowledge and safer practices if they had received infection prevention training and had infection prevention guidelines in their workplace. Interventions should be designed to consider these identified factors.

## Background

Infection prevention plays a key role in preventing and reducing the rate of healthcare associated infection (HAIs). HAIs, are the most frequent adverse event in healthcare worldwide can occur as a part of an endemic or epidemic situation and affect the quality of care of hundreds of millions of patients every year in both developed and developing countries [1, 2]. According to the Centers for Disease Control and Prevention (CDC), HAIs defined as infections localized or systemic condition resulting from adverse reaction to the presence of infectious agent or its toxins acquired from health care settings that was not incubating or symptomatic at the time of admission to the healthcare facility [3]. These infections are a major public health concern and a threat to patient safety, contributing to increased morbidity, mortality, and cost [2, 4]. Based on the available evidence, the overall impact of HAIs implies prolonged hospital stay, long-term disability, increased resistance of microorganisms to antimicrobials, high costs for patients and their family, and unnecessary deaths [5–7]. In addition, it places a significant massive additional economic burden on the health care system [8].

According to World Health Organization (WHO), of every 100 hospitalized patients, 10 in developing countries and 7 in developed countries will acquire at least one HAI [9]. The CDC also estimates that 2 million patients suffer from HAIs every year and nearly 100,000 of them die in United States (US) [10]. In US and Europe the point prevalence of patients with at least one HAI in acute care hospitals has reached 6%, prevalence (19.5%) was highest among patients admitted to intensive care units (ICU) [11, 12]. By contrast in developing countries, the problem is three times higher when compared to the incidence observed in adult intensive care units in the US [1]. It is also thought that the prevalence is more than 40% in parts of Asia, Latin America and sub-Saharan Africa [13, 14]. In sub-Saharan Africa lone, the rate of HAIs ranges from 2.5 to 30.9% with patients undergoing surgery, the most frequently affected [15–17]. This high proportion of surgical site infection is also seen in studies conducted in Ethiopia, with the prevalence ranging from 11.4 to 52.1% [18–21].

The high burden of HAIs in Ethiopia as well as in many developing countries has been reported to be higher because of the large number of patients, the limited number of staff, and insufficient compliance with infection prevention and control measures [22–25]. Strict adherence to infection prevention protocol is critical to avoiding spread of infection among hospitalized patients and fundamental to quality of care [14, 25–27]. Infection prevention programs, including campaigns to improve hand hygiene, are effective in reducing HAIs [28]. Even a small improvement in hand hygiene compliance by 10%, was associated with a 6% reduction in overall HAIs and 14% reduction in healthcare-associated *Clostridium difficile* infection [29]. In support of this, effective implementation of infection prevention practices in healthcare facilities leads to a significant reduction more than 30% in HAIs [30]. The financial impact of infection prevention practices is also estimated to be $25.0 billion to $31.5 billion in medical cost savings in US [31, 32].

There is little evidence concerning the burden of unsafe care and infection prevention practice in resource limited settings [19, 33–37]. Although most HAIs can be prevented with relatively inexpensive infection prevention and control measures such as hand washing, studies have shown that healthcare facilities in Africa do not have effective infection control programs [13, 14, 24, 25].

In Ethiopia, different activities have been made relentlessly by Federal Ministry of Health of Ethiopia to scale up infection prevention program and to put together all up-to-date information and practical interventions in the area of infection prevention and patient safety as a healthcare reform initiative [13, 25]. Despite of this efforts, infection prevention activities is low [37–39] and high burden of HAIs in Ethiopia is a great concern [18–21]. In addition to this, there is limited national data on infection prevention regardless of the dramatic increase in the development of healthcare facilities. Few studies have been conducted and the majority of them are case studies limited to a few healthcare facilities in close proximity to each other [37–44]. In the study area of southwest Ethiopia, to our knowledge, no studies have been undertaken on infection prevention knowledge and practice among healthcare workers. The first step to developing a successful infection control program is to undertake an assessment of existing infection prevention practice [35]. Therefore, this study aimed to assess the knowledge and practice of healthcare workers towards infection prevention and associated factors in healthcare facilities in southeast Ethiopia. The present study will be essential for policy and decision makers in the development of HAIs prevention programs, and strategic plans. The finding also helps healthcare workers to improve the quality of healthcare delivery services and infection prevention activities.

## Methods

### Study design, setting and population

A facility-based cross-sectional study was conducted from April 6 to 10, 2015 in public healthcare facilities in West Arsi District, Southeast Ethiopia. According to the West Arsi District Health Department Biannual Healthcare Workers Profile Report, there are a total of 2175 fulltime healthcare workers working in eighty-one healthcare centers, two primary hospitals and one general hospital. All healthcare workers working in the healthcare facilities who provide care and have direct involvement in patient care were eligible to be included in the study. These workers were physicians, health officers, nurses, midwives, anesthetists, laboratory technicians, laboratory technologists, pharmacists, pharmacy technicians, environmental health officers, and radiographer. Individuals who were on annual and maternity leave during data collection time and those who could not respond to the questions due to illness were excluded from the study.

### Sample size determination

The sample size was determined using the single population proportion formula. It was computed by considering that previous study has demonstrate that 84.2% of the respondents demonstrated good infection prevention knowledge and 54.2% demonstrate safe practices towards infection prevention [44]; a 95% confidence level, and 5% margin of error. The largest sample size was considered (*n* = 381). A finite population correction formula was considered since the source population is less than 10,000. Accordingly, the required total sample size was 680, after taking a design effect of 2 and 5% non-response rate.

### Sampling procedure

A multi-stage sampling technique was used to select study participants. First, all public healthcare facilities in West Arsi District were stratified based on their level of service delivery as general hospital, primary hospital and health center [45]. Then, one general hospital, one primary hospital, and 28 health centers were randomly selected using a lottery method. The calculated sample sizes (*n* = 680) were allocated proportional to each selected facilities. Finally, healthcare workers were selected by using a lottery method from the list of healthcare workers obtained from each facility (117 selected from the general hospital, 72 from the district hospitals and 491 from health centers).

### Variables and measurements

The dependent variables studied were knowledge and practice of healthcare workers towards infection prevention. Whereas, the independent variables include sex, profession, educational level, year of service, presence of infection prevention committee, availability of infection prevention guidelines in the working department, training about infection prevention, and availability of water in working department.

Healthcare workers’ knowledge regarding infection prevention was measured by ten “yes or no” questions. A scoring system was used in which the respondent’s correct and incorrect answers provided for the questions were allocated “1” or “0” points respectively. Knowledge scores were summed up to give a total knowledge score for each healthcare worker. The total score of knowledge questions ranging from 0 to 10 were classified into two categories of response: knowledgeable (if above the mean) and not knowledgeable (equal to or below the mean) [37, 44].

The healthcare workers infection prevention practice was measured by ten items in which responses were answered in a three point Likert scale (always or yes, sometimes, never) options. To analyze the practice, similar procedures were followed a score of 1 was assigned for each acceptable or correct practice and 0 for unacceptable, hence the total score of infection prevention practice ranged from 0 to 10. Accordingly, healthcare workers infection prevention practice was classified into two categories: safe (if above the mean) and unsafe (equal to or below the mean) [37, 44].

### Data collection procedures and quality control

A pre-tested structured self-administered questionnaire was used to collect data. The data collection tool was developed by reviewing relevant literature [13, 14, 25] and by adapting the content from related studies [40, 44]. The data collection tool was first prepared in English, translated to Afan Oromo (the local language) then retranslated to English to check for consistency. Data collection was facilitated by five trained nurses and two supervisors. To enhance instrument reliability, the instrument was pre-tested on 34 individuals (5% of the intended sample size drawn from outside of the study area in nearby healthcare facilities of Bale Zone with similar characteristics to those in the study). In addition, to improve the validity of the questions the tool was checked by two experts in the field of infection prevention; based on their comments corrections were made before data collection.

The data collection tool was a three-part questionnaire. The first part of this questionnaire included the background and demographic features of healthcare workers (age, sex, marital status, profession, educational level, year of service, history of infection prevention training, and the presence of infection prevention guidelines in their department). The second part consists of ten questions concerning knowledge about infection prevention on the following topic: general awareness regarding infection prevention, personal protective equipment (PPE), hand washing, alcohol based hand antiseptic, tuberculosis (TB) infection control measures, medical instrument decontamination, healthcare waste handling, and infections transmitted through needle stick injuries. The third part consisted of ten questions self-reporting infection prevention practices in the areas of PPE utilization, hand hygiene, instrument processing, healthcare waste handling, and safe injections. The data collection process which occurred over a five day period was checked by two supervisors on daily basis. Questionnaires were checked for completeness and consistency. The data collection tool was tested for internal consistency (reliability) using Cronbach’s alpha test. The resulting Cronbach’s alpha values were 0.812 and 0.751 for the knowledge and practice sections, respectively.

### Data processing and analysis

Data were entered into Epi-Info version 3.5.1 software and exported to SPSS version 21 for analysis. Descriptive statistics were used to present the frequency distribution of important variables. For the purposes of analysis, the dependent variables were dichotomized into binary outcome variable indicating; “infection prevention knowledge” was coded as “knowledgeable = 1” and “not knowledgeable =0” and “infection prevention practice” coded as “safe = 1” and “unsafe = 0”. Initially, bivariate analyses were performed to assess association between the dependent and independent variables and those variables with a *p*-value of < 0.25 were then entered into multivariable logistic regression to control the effect of confounder’s and to estimate the independent predictors of infection prevention knowledge and practice [46]. A regression model was built by stepwise logistic regression procedure. Predicting power of variables in the final fitted model was checked by receiver observed characteristics (ROC) curve. The Hosmer and Lemeshow test was used for overall goodness of fit [47]. Odds ratios with 95% confidence intervals were used to determine the strength of association between the dependent and independent variables. All tests were two-tailed and *p*-value < 0.05 was used as a cut-off point for all statistical significant tests.

## Results

### Socio-demographic characteristic of healthcare workers

A total of 648 healthcare workers participated in the study, for a response rate of 95.3%.The mean (standard deviation) age of healthcare workers was 28.23(±5.2) years. Four hundred forty-six (68.8%) of participants were male. The majority of them (61.4%) were nurses (Table 1).

**Table 1:** Socio-demographic characteristic of healthcare workers in healthcare facilities of West Arsi District, Southeast Ethiopia, April 2015 (*n* = 648)

Variables	Category	Number	Percent
Age	18–29	345	53.2
	30–39	262	40.4
	40–49	36	5.6
	≥ 50	5	0.8
Sex	Male	446	68.8
	Female	202	31.2
Marital status	Single	349	53.9
	Married	284	43.8
	Divorced	13	2.0
	Widowed	2	0.3
Profession	Nurse	398	61.4
	Midwife	104	16
	Health Officer	51	7.9
	Physician	22	3.4
	Laboratory technicians and others ♣	73	11.3
Educational level	Diploma	394	60.8
	First degree	242	37.3
	Second degree & above	12	1.9
Year of service	< 5 years	390	60.2
	5–9 years	170	26.2
	10–14 years	48	7.4
	≥ 15 years	40	6.2
Ever taken training on infection prevention	Yes	184	28.4
	No	464	71.6
Presence of infection prevention committee	Yes	430	66.4
	No	218	33.6
Availability of infection prevention guidelines in the working department	Yes	310	47.8
	No	338	52.2

♣ Pharmacist, pharmacy technicians, anesthetist and environmental health officers

### Knowledge about infection prevention

In this study, only 348 (53.7%) [95%CI: 49.8, 57.4%] of the respondents found to be knowledgeable about infection prevention (Table 2).

**Table 2:** Healthcare workers knowledge regarding infection prevention in healthcare facilities of West Arsi District, Southeast Ethiopia, April 2015 (*n* = 648)

Knowledge items	Number^a^	Percent
I have heard about infection prevention principles	575	88.7
Gloves cannot provide complete protection against transmission of infections	385	59.4
Washing hands with soap or use of an alcohol based antiseptic decreases the risk of transmission of healthcare acquired infections	429	66.2
Use of an alcohol based antiseptic for hand hygiene is as effective as soap and water if hands are not visibly dirty	422	65.1
Gloves should be worn if blood or body fluid exposure is anticipated	403	62.2
Hand washing is necessary before procedures are performed	300	46.3
Tuberculosis (TB) is carried in airborne particles that are generated from patients with active pulmonary tuberculosis	471	72.7
There is no need to change gloves between patients as long as there is no visible contamination	248	38.3
Do you know how to prepare 0.5% chlorine solution?	365	56.3
Safety box should be closed/sealed when three quarters filled	318	49.1

^a^Healthcare workers “Yes” response

### Self-reported infection prevention practice

In this study, the proportion of healthcare workers who reported safe infection prevention practice was found to be 235(36.3%) [95%CI: 32.4,40.1%] (Table 3). Four hundred fifty (69.4%) reported that they frequently wash their hands after patient care, 416(64.2%) after removing gloves, 412 (63.6%) before care of wounds, and 364 (56.1%) before patient care.

**Table 3:** Infection prevention practice of healthcare workers in healthcare facilities of West Arsi District, Southeast Ethiopia, April 2015 (*n* = 648)

Practice items	Response	Number	Percent
Do you apply antiseptic hand rub to clean hands?	Yes	403	62.2
	No	245	37.8
Did you practice high-level disinfection where sterilization is not applicable?	Yes	377	58.2
	No	271	41.8
Do you use all Personal Protective Equipment’s (PPE) to prevent the risk of acquiring and/or transmitting infection?	Yes	390	60.2
	No	258	39.8
Did you mix dry and liquid healthcare wastes?	Yes	443	68.4
	No	205	31.6
Do you incinerate or bury used sharp materials?	Yes	388	59.9
	No	260	40.1
When do you change disinfectant chlorine solutions?	Every 24 h	351	54.2
	Every two days	174	26.8
	Immediately when it is soiled	0	0
	I don’t know	123	19.0
For how long do you soak reusable medical instruments in chlorine solution?	10 min	375	57.9
	1 h	203	31.3
	24 h	40	6.2
	5 min	30	4.6
How often do you use glove (both hands)?	Always	339	52.3
	Sometimes	303	46.8
	Never	6	0.9
Do you wear the necessary personal protective equipment (PPE) such as gloves, apron, goggles and mask, if splashes and spills of any body fluids are likely?	Always	253	39.0
	Sometimes	381	58.8
	Never	14	2.2
Where do you usually put sharp disposal boxes?	In high traffic area	153	23.6
	At corridor	394	60.8
	Any where	101	15.6
	Hand reach area	0	0

### Healthcare workers occupational exposure status

The life-time prevalence of self-reported needle stick injury and blood or body fluid exposure was 210 (32.4%) [95% CI: 28.7, 36.1%] and 253 (39.0%) [95% CI: 35.2, 43.1%] respectively. Among healthcare workers who reported needle stick injury, 131 (62.4%) were injured once, 52(24.8%) reported two injuries and 27(12.9%) were injured three or more times. Healthcare workers received needle stick injuries while securing intravenous catheters 58(27.62%), during recapping 72(34.29%) during suturing 110(52.38%), during the handling of healthcare waste 9(4.29%) and during blood sample taking 10(4.76%). Respondents indicated that the disease transmitted by needle stick injury were Human Immunodeficiency virus (HIV) 636(98.1%), Hepatitis B virus (HBV) 511(78.9%), Hepatitis C virus (HCV) 302(46.6%), and Tuberculosis (TB) 7(1.1%).

### Factors associated with knowledge of healthcare workers towards infection prevention

In the bivariate analysis, sex, profession, service year, presence of infection prevention committee, presence of infection prevention guideline, and ever taking training on infection prevention were factors which were significantly associated with knowledge about infection prevention. However, only profession, service year, presence of infection prevention committee, presence of infection prevention guideline, and ever taking training on infection prevention were found to be significantly associated in the multivariable logistic regression model.

Physicians were 85% less knowledgeable on infection prevention than nurses [AOR (Adjusted Odds Ratio) = 0.15, 95% CI: 0.05, 0.45]. Those healthcare workers who have served for ten and above years were about 3.41 times more likely knowledgeable about infection prevention than those whose service years are less than five years (AOR = 3.41, 95% CI: 1.22, 9.55). Those healthcare workers in facilities with infection prevention committees were about 1.78 times more likely to be knowledgeable about infection prevention than their counterparts (AOR = 1.78, 95% CI:1.01, 3.13). Those healthcare workers who have infection prevention guidelines in their working department were about 2.44 times more likely to be knowledgeable about infection prevention than those who don’t (AOR = 2.44, 95% CI: 1.45, 4.12). Those healthcare workers who have ever taken training on infection prevention were about 5.02 times more likely to be knowledgeable about infection prevention than those who have not (AOR = 5.02, 95% CI:1.45,8.59) (Table 4). The study also identified a strong linear correlation between healthcare workers infection prevention knowledge score and the practice score (Pearson correlation coefficient = 0.703, *p* < 0.001).

**Table 4:** Factors associated with healthcare workers infection prevention knowledge in healthcare facilities of West Arsi District, Southeast Ethiopia, April 2015 (*n* = 648)

Variables	Category	Infection prevention knowledge status		Crude OR (95% CI)	Adjusted OR (95% CI)
		Knowledgeable (*n* = 348)	Not-Knowledgeable (*n* = 300)		
Sex	Male	182	264	0.15(0.09,0.22)*	
	Female	166	36	1	
Profession	Nurse	197	201	1	1
	Midwifery	68	36	0.52(0.33,0.81)*	0.49(0.24,1.02)
	Physician	17	5	0.29(0.10,0.79)*	0.15(0.05,0.45)**
	Health Officers	29	22	0 .74(0.41,1.34)	0.54(0.21,1.38)
	Laboratory technicians and other♣	37	36	0.95(0.58,1.57)	0.94(0.47,1.90)
Educational level	Diploma	201	193	0.75(0.55,1.04)	
	First degree and above	147	107	1	
Year of service	< 5 years	212	178	1	1
	5–9 years	92	78	1.39(0.72, 2.74)	1.01 (0.59, 1.71)
	≥ 10 years	44	44	1.41(0.69, 2.87)	3.41(1.22, 9.55)**
Presence of infection prevention committee	Yes	281	149	1	1
	No	67	151	2.35 (0.17, 0.33)*	1.78(1.01,3.13)**
Availability of infection prevention guidelines in the working department	Yes	231	79	5.52(3.93,7.76)*	2.44(1.45, 4.12)**
	No	117	221	1	1
Ever taken infection prevention training	Yes	174	10	3.56(1.49,5.64)*	5.02(1.45, 8.59)**
	No	174	290	1	1

*OR* Odds Ratio *(*P < 0.05*) crude, ** (*p* < *0.05*) adjusted, ♣Pharmacist, pharmacy technicians, anesthetist and environmental health officers

### Factors associated with healthcare workers infection prevention practice

In the bivariate analysis, sex, profession, service year, availability of water for hand washing in the healthcare worker’s ward or department, the presence of an infection prevention committee, availability of infection prevention guidelines, and ever having taken taking training on infection prevention were factors which were significantly associated with healthcare workers’ infection prevention practice. However, only profession, the presence of infection prevention guidelines, and having ever taken ever taking training on infection prevention were found to be significantly associated in the multivariable logistic regression analysis.

Midwives were about 72% times less likely to use safe infection practices as compared to nurses (AOR = 0.28, 95% CI: 0.12, 0.69). Healthcare workers who have infection prevention guidelines available were 3.34 times more likely to practice safely infection prevention compared to those who do not have guideline for their practice (AOR = 3.34, 95% CI: 1.65, 6.76). In addition, healthcare workers who have ever taken training on infection prevention were about 5.31 times more likely to practice safe infection prevention than those who have not received training (AOR = 5.31, 95% CI: 2.42, 11.63) (Table 5).

**Table 5:** Factors associated with healthcare workers infection prevention practice in selected healthcare facilities of West Arsi District, Southeast Ethiopia, April 2015 (*n* = 648)

Variables	Category	Infection prevention practice status		Crude OR (95%CI)	Adjusted OR (95%CI)
		Safe (*n* = 235)	Unsafe (413)		
Sex	Male	92	354	0.11(0.07,0.15)*	
	Female	143	59	1	
Profession	Nurse	134	264	1	1
	Midwifery	44	60	0.37(0.24,0.58)*	0.28(0.12, 0.69)**
	Physician	19	3	3.22(0.94,11.06)	2.99(0.25, 36.08)
	Health officers	17	34	1.02(0.55,1.88)	0.44(0.15, 1.34)
	Laboratory technicians and other♣	21	52	1.26(0.73, 2.17)	2.83(0.83, 9.60)
Educational level	Diploma	143	251	1.00(0.72, 1.39)	
	First degree and above	92	162	1	
Year of service	< 5 years	147	243	1	1
	5–9 years	61	109	1.08(0.74,1.57)	0.57(0.18, 1.83)
	10-14 years	11	37	2.05(1.01,4.11)*	0.49(0.15, 1.67)
	≥ 15 years	16	24	0.91(0.47,1.76)	3.17(0.47,21.24)
Availability of water in working department	Yes	203	318	1	1
	No	32	95	1.89(1.22, 2.94)*	0.35(0.06, 1.96)
Presence of infection prevention committee	Yes	199	231	1	1
	No	36	182	4.36(2.91,6.53)*	1.204(0.55, 2.62)
Availability of infection prevention guidelines in the working department	Yes	167	143	4.64(3.28, 6.56)*	3.34(1.65, 6.76)**
	No	68	270	1	1
Ever taken infection prevention	Yes	126	58	7.08(4.85, 10.32) *	5.31(2.42, 11.63) **
	No	109	355	1	1

*OR* Odds Ratio *(*P* < 0.05) crude, ** (*p* < 0.05) adjusted, ♣ Pharmacist, pharmacy technicians, anesthetist and environmental health officers

## Discussion

Reducing the risk of HAIs and using infection prevention principles are in the control of healthcare workers; therefore, healthcare workers must have correct, up-to-date and appropriate scientific information and practice accordingly [48]. Without adequate infection prevention and patient safety practices both healthcare workers and patients are at risk of acquiring serious infections such as HIV, HBV, HCV, and Methicillin Resistant *Staphylococcus aureus* (MRSA) infection as well as other bacterial and viral infections [13, 49–51]. Recent studies also suggest that proper and consistent application of existing infection prevention and control practices can lead to up to a 70% reduction in certain HAIs [52, 53].

In this study, the proportion of healthcare workers who were knowledgeable about infection prevention was found to be 53.7%. This finding indicated that a large percentage of respondents (46.3%) in the healthcare facilities studied demonstrated inadequate knowledge about infection prevention, a finding in line with similar studies in Ethiopia [37, 39, 50] and in Africa [54]. On the other hand, the proportion of knowledgably participants is lower than studies in facilities in Bahir Dar city and Addis Ababa which reported 69% and 84.2% of healthcare workers had good infection control knowledge [40, 44]. This discrepancy may be due to difference in study setting and study variables since the former study focused only the two components of infection prevention (hand hygiene and tuberculosis infection control) and includes only two university hospitals in Addis Ababa the later includes private healthcare facilities. Similarly, the result is inconsistent with that of Abdella et al. [41], who found that 77.3% of the respondents were knowledgeable on hand hygiene compliance, Gizaw et al. [43], who reported 63.9% of the respondents had good knowledge on tuberculosis infection control, and Shrestha et al. [55], who also found that more than half (54%) of healthcare workers had good level of knowledge on tuberculosis infection control in Nepal. The variation in the percentage can be attributed to different methodological approaches and sample healthcare facility dissimilarity where in the previous studies they only assess a single infection prevention component like hand hygiene compliance and tuberculosis infection control while our study also included other infection prevention components such as medical instrument disinfection, personal protective equipment use, and healthcare waste handling.

The implication of the finding suggested that healthcare workers in studied public healthcare facilities lack evidence-based knowledge and appropriate scientific information about infection prevention. The possible reason for lower finding in the current study might be due to lack of training about infection prevention, in this study only 28.4% of healthcare workers received infection prevention training.

As evidenced from the result of multivariable logistic regression analysis of this study and many related studies from Ethiopia, Italy, Nepal and Nigeria reported infection prevention knowledge of healthcare workers was positively associated with training [43, 50, 51, 55–57]. This could be due to the fact that updating the knowledge of the health workers about infection prevention principles could have changed the older understanding and could have resulted in good score on knowledge questions [50]. The finding highlights the necessity of infection prevention training in the improvement of healthcare workers knowledge.

This study showed that physicians are less knowledgeable about infection prevention than nurses. The result is consistent with Parmeggiani et al. [51] in Italy, who found that nurses were more likely to have greater knowledge than physicians on the use of standard precautions and hand hygiene to control HAIs. Inconsistent results were reported by Alkubati et al. [58], who reported knowledge about prevention of central venous catheter-related infection was not significantly different between physicians and nurses in Egypt’s Alexandria University hospital. Difference in knowledge level of health workers about infection prevention could be due to dissimilarity in training and awareness about infection prevention. Additionally, nurses are in the forefront of patients care in healthcare facilities, which could help them to have better knowledge.

This study also suggests that years of service of the study participants are significantly associated with knowledge about infection prevention. Healthcare workers who have served for ten and more years were about three times more likely to appear knowledgeable about infection prevention than those with less than five years service. This finding is in line with other related studies from Ethiopia [43, 44, 50], Africa [59], Europe [60], and Asia [48, 52], in which years of service year were positively associated with knowledge regarding infection prevention. The strong positive association from this study could be due to the fact that as the number of years of service increases, healthcare workers are repeatedly exposed to infection prevention principles and became more experienced and knowledgeable.

The presence of a positive linear correlation existed between healthcare workers total knowledge score and practice (Pearson correlation coefficient = 0.703, *p* < 0.001) is also in agreement with studies conducted in Ethiopia and elsewhere [43, 52]. As a result with improved knowledge, practice can be also improved.

In this study, the proportion of healthcare workers who appear to be practicing safe infection prevention practice was 36.3%. This result is much lower than with many similar studies in Ethiopia [37, 39, 42–44, 50]. This may be explained by the fact that the vast majority healthcare workers in the study area (71.6%) had not received infection prevention training and had inadequate infection prevention knowledge. As well (60.2%) of these workers had less than five year’s work experience. Our study may have indicated a gap in training which could result in poor infection prevention practice among healthcare workers.

The current finding is lower than a study done in an Egyptian hospital where 57.1% of the health workers were found to practice satisfactory infection prevention activities [61]. This could be due to differences in study setting, study variables, a difference in the definition of satisfactory practice and other methodological concerns. Difference in knowledge of the healthcare workers concerning infection prevention could be another factor for this inconsistency. However, the findings here are better than those findings from studies done in Iran where only 32.1% of healthcare workers reported moderately-good compliance in hand hygiene [52]. Similarly, the finding is better than reported by Abdella et al., from Ethiopia which reported healthcare providers hand hygiene compliance of 16.5% in Gondar University Hospital [41].

The low percentages of healthcare workers adherence to infection prevention principles in the present study may be explained by factors suggested by the questions posed to workers. On one hand, poor baseline knowledge of infection prevention principles may contribute importantly. On the other hand, other factors such as lack of supportive supervision from an infection prevention committee, and other organizational supports may be lacking.

The present study also found out significant differences in the practice of infection prevention among healthcare workers who had infection prevention training and have infection prevention guideline in their working department. The odds of safe practice were likely to be three and five times higher in healthcare workers who had infection prevention guidelines available and trained in infection prevention respectively. This finding is in agreement with other similar studies in Ethiopia [37, 41–43, 50, 57] and elsewhere [51].

Another factor which was significantly associated with safe infection prevention practice is profession. This study found out differences in the reported practices of infection prevention among different healthcare professionals, such as the odds of safe practice among midwifes likely to be reduced by 72% compared to nurses. Other research by Biniyam et al. [37] has reported dissimilar infection prevention practices between physicians and laboratory technicians in Ethiopia, and between nurses and physicians by Parmeggiani et al. [51] in Italy. This could be due to difference in training and operational definition of the practice from study to study. Variation in job description of different health professionals may be another factor for this discrepancy.

Year of service was not found to be statistically significant on multivariable analysis in this study. However, in the bivariate analysis the odds ratio suggests that healthcare workers who have higher ten and above service year were about two times more likely to had safe practice when compared with those who had less than five. In support of this, Hosseinialhashemi et al. [52] from Iran, reported a correlation between hand hygiene practice and work experience (*p* < 0.05).

The current study also detected a potential high prevalence of occupational exposure to needle stick injury and blood and body fluid splashes among healthcare workers in the study area, which is similar to other related studies in Ethiopia [37, 38]. The problem highlights the need to improve safe infection practice across healthcare facilities.

This study has several limitations; due to the cross-sectional nature of this study design temporal relationships cannot be established between the explanatory and outcome variables of infection prevention knowledge and practice. Despite, the high response rate in this study, social desirability bias and recall bias are potential limitations of these self-reported results. Healthcare workers might not give true and genuine responses on the self-administered questionnaire, preferring to provide more socially acceptable responses than their actual day to day practice. Lack of standardized questionnaires with acceptable reliability and validity for assessing infection prevention knowledge and practice in Ethiopia was another limitation of the study that limits our findings. To overcome this problem we included items that are acceptable face-validly and reliability, used by other authors in order to aid comparison. One additional limitation of this study is that the generalization of findings limited to public healthcare facilities.

## Conclusion

The present study revealed that a significant proportion of healthcare workers were not knowledgeable about infection prevention. The overall level of safe infection prevention practice among healthcare workers is considered to be very low. The current study also detected that there was a high prevalence of occupational needle stick injury and blood and body fluid splashes among healthcare workers. Factors such as the presence of infection prevention guidelines in the work place and training were independent predictors of safe infection prevention practice and better knowledge. Providing on job continuous educational training on infection prevention is essential as well as ensuring the availability of infection prevention guidelines in working department should be effective and important interventions to improve healthcare workers infection prevention practice and knowledge. In the future researchers should consider stronger observational study designs to validate the self-reported practice of healthcare workers and to determine actual practices, as well as the actual prevalence of HAIs as result of poor infection prevention practice.

## Abbreviations

AOR: Adjusted odds ratio
CDC: Centers for disease control and prevention
CI: Confidence interval
COR: Crude odds ratio
HAIs: Healthcare associated infections
HBV: Hepatitis B virus
HCV: Hepatitis C virus
HIV: Human immunodeficiency virus
IRB: Institutional review board
PPE: Personal protective equipments
SPSS: Statistical package for social sciences and
TB: Tuberculosis
WHO: World Health Organization
